# Evaluation of a validated questionnaire to assess the need for prevention or rehabilitation by preventive health examinations: a cross-sectional study of German employees aged 45 to 59 years (Ü45-check)

**DOI:** 10.3389/fpubh.2025.1480312

**Published:** 2025-07-16

**Authors:** Linda Kalski, Tilman J. Pulst Caliman, Franziska Greiß, Athanasios Karathanos, Lorena Hafermann, Laura Völkel, Charleen Pächter, Carolin Herrmann, Maja A. Hofmann, Bernd Wolfarth

**Affiliations:** ^1^Institute of Sport Science, Humboldt-Universität zu Berlin, Berlin, Germany; ^2^Department of Sports Medicine, Charité – Universitätsmedizin Berlin, Berlin, Germany; ^3^Institute of Biometry and Clinical Epidemiology, Charité – Universitätsmedizin Berlin, Corporate Member of Freie Universität Berlin and Humboldt-Universität zu Berlin, Berlin, Germany; ^4^Mathematical Institute, Heinrich Heine University Düsseldorf, Düsseldorf, Germany; ^5^Department of Dermatology and Venereology, Charité – Universitätsmedizin Berlin, Berlin, Germany; ^6^Federal German Pension Insurance, Berlin-Brandenburg, Berlin, Germany

**Keywords:** screening, questionnaire, prevention, rehabilitation, work disability, disability pension, Ü45-check

## Abstract

**Objective:**

A longer life expectancy can lead to a longer working life and is therefore important for the individual and the healthcare system. Therefore, a study group established a Risk-Index Disability-Pension (RI-DP), which assesses the risk of work disability. However, a standardized and well-founded preventive health examination does not yet exist in Germany. Hence, we developed a preventive health examination conducted by physicians and compared it with a questionnaire survey to examine its differences and results in relation to the need for prevention and rehabilitation, taking into consideration the RI-DP.

**Methods:**

In this prospective cross-sectional study, *n* = 1,040 participants (45–59 years) took part in a preventive health examination at the Charité - Universitätsmedizin Berlin/Humboldt-Universität zu Berlin, Germany. A questionnaire survey and preventive health examination, including anthropometric measures, anamnesis, cardiovascular examinations, and blood samples, were conducted independently to determine the need for prevention and rehabilitation.

**Results:**

The mean age was 52.93 (sd = 4.17) years, and *n* = 631 (61%) were men. The questionnaire assessed *n* = 733 (70%) participants as needing no additional action (green), *n* = 215 (21%) as needing a prevention program (yellow), and *n* = 91 (9%) as needing a rehabilitation program (red). In contrast, physicians assessed *n* = 141 (14%) as ‘green’, *n* = 717 (69%) as ‘yellow’, and *n* = 181 (17%) as ‘red’, revealing substantial discrepancies, especially in preventive needs. The leading associations of individual factors on physicians’ evaluation were BMI [OR 1.135 (1.094; 1.178)], SBP [OR 1.099 (1.009; 1.197)], smoking status [OR 1.691 (1.212; 2.366)], depressive moods [OR 2.254 (1.565; 3.254)] and physical activity 1-2 h/week [OR 0.618 (0.436; 0.874)], and on the questionnaire: gender male [OR 1.790 (1.057; 3.046)], and depressive moods (OR 4.506 [3.216; 6.322]). This underlines the complementary nature of the two approaches in evaluating health interventions. According to the RI-DP, the participants had the following risk of early retirement: low-risk *n* = 540 (52%), medium-risk *n* = 307 (30%), and high-risk *n* = 193 (19%).

**Conclusion:**

The results emphasize the need for combining questionnaire surveys with preventive health examinations to assess health needs comprehensively. Notably, the preventive health examination suggests higher prevention needs compared to the questionnaire, indicating that the questionnaire may not fully capture clinical prevention needs.

**Clinical trial registration:**

https://drks.de/search/de/trial/DRKS00030982, identifier DRKS00030982.

## Introduction

In comparison to previous generations, individuals are now more likely to live to an advanced age, with life expectancy increasing to 80 years or more (1). Such changes may consequently result in an increased pension age. Therefore, it is of utmost importance to prioritize employees’ well-being and cultivate a healthy and ergonomic work environment. Similar trends have been observed globally. For example, studies conducted in Sweden and Japan have examined the implications of increasing life expectancy on workforce participation and retirement policies. These studies emphasize the need for adaptations in occupational health and pension systems (2, 3).

It is commonly agreed that the demand for rehabilitative care and participation has increased in the current century (4, 5) and must be strengthened to supply an effective rehabilitation system (6, 7). Regarding the general research and investigation on rehabilitation, the medical benefits of rehabilitative programs are well known. Next to the medical concern regarding rehabilitation governments, and health systems also have a financial interest. Due to the amount of money health systems spend on rehabilitative measures, the treatment results of the checkups must be reliable and effective. A treatment resulting in no improvement for the patient is a financial burden for the healthcare system, patients, and the government because the costs are usually high.

In response to the recent trend toward flexible pensions across the European Union (8), the German government passed the so-called “Flexirentengesetz” (flexible pension law) in 2016, which allows German citizens to determine their retirement age more individually (9). The objective of the legislation is to facilitate a more flexible transition between employment and retirement while also providing incentives for longer periods of employment. This results in the potential for additional income opportunities when working longer and fewer restrictions when retiring early. Individuals have the option of claiming their old-age pension at an age between 63 and 67, contingent upon the number of years worked and the individual’s birth year (10). For each additional year of employment after the regular individual retirement age, individuals receive a 6% increase in their pension per year (11).

The flexible pension law and retirement system reinforce the importance of prevention and rehabilitation in the workplace. This necessitates the establishment of a stable health system. Interventions are implemented to preserve work capacity and promote health to facilitate the transition into retirement and to ensure the secure payment of pensions. A preventive health examination has the potential to identify possible future health issues, thereby positively influencing an individual’s health status. Consequently, the likelihood of developing adverse health outcomes can be reduced, thereby maintaining an individual’s overall health status.

Preventive screening programs targeting work ability and early retirement risks have been introduced in other countries. For instance, Finland has implemented a workplace survey to detect early signs of occupational health risks. The results of this survey have indicated positive outcomes with regard to labor retention (12).

In 2011, the study group by Bethge et al. published a risk level prediction index, the so-called Risk Index – Disability Pension (RI-DP) (13, 14). The RI-DP identified pertinent prognostic variables pertaining to work disability; however, it does not measure guidance regarding the most current and necessary prevention and/or rehabilitation measures. Consequently, empirically proofed preventive health examinations and questionnaires are required to assess these demands.

In Germany, various preventive examinations are offered. Most of them specialize in the early detection of cancer (e.g., breast cancer, prostate cancer, skin cancer, and colorectal cancer). Furthermore, people between the ages of 18–35 years can participate in a one-time health check-up which includes an anamnesis and physical examination for early detection of kidney disease, cardiovascular disease, diabetes, and hepatitis B and C virus infections. When reaching the age of 35 years, the check-up can be utilized every three years. Up to now, possible risk factors for early retirement or limited health prognostics are not included in the check-ups (15, 16).

Based on the arguments described above and the objective of the coalition agreement for the years 2021–2025, a screening survey was developed to determine the need for prevention or rehabilitation in people over 45 years of age (Ü45-Check). The comprehensive study design, including detailed descriptions of the methodology, recruitment strategies, inclusion criteria, and planned analyses, has already been published in a separate protocol paper (17).

The objectives of this study are the following: (1) to determine the need for prevention or rehabilitation based on preventive medical health examinations compared to a questionnaire survey and (2) to examine the association of items of the medical examinations, the questionnaire, and the RI-DP on a potential disability pension. In the future, the “Ü45-Check” can be used as a fundamental check-up tool to support health despite a potentially later retirement age. As a long-term goal, the Ü45-Check could be implemented in general practices to further increase and secure participation and medical services for people because authorized access is just as important as the need itself.

The explicit study questions underlying this article are as follows:

1. Are there differences in the assessment of the need for prevention or rehabilitation based on preventive health examinations compared to assessing the need for preventive or rehabilitation measures by a questionnaire survey?
2. Is there any relation between the results of the preventive health examinations and the RI-DP?
3. Is there any relation between the results of the questionnaire survey and the RI-DP?

## Methods

### Study design and population

**Figure 1:**
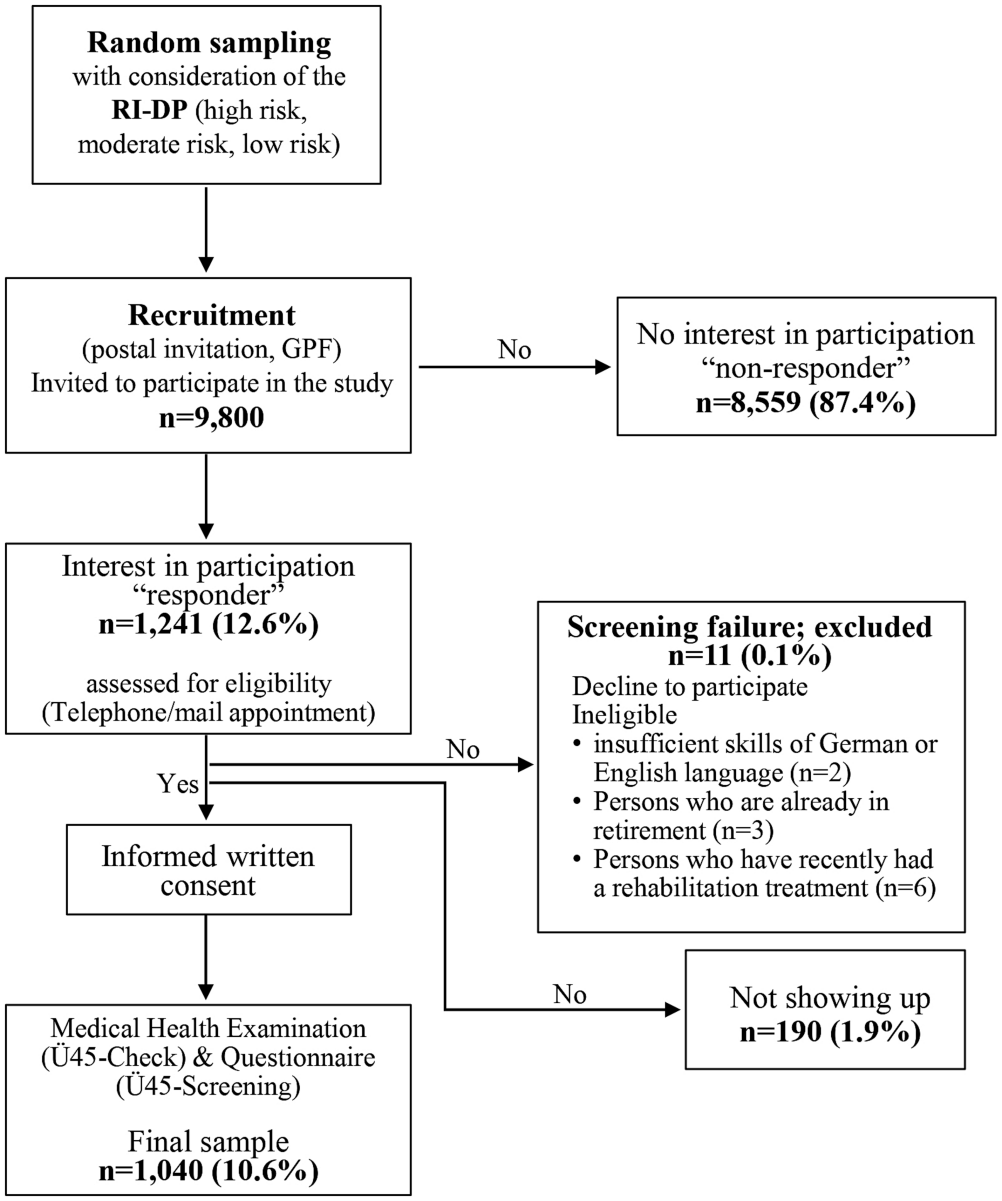
Flow Chart of recruitment of participants included in the Ü45 trial form 2021 until 2024. The reasons for the screening failure were, e.g., an ongoing procedure regarding a claim for a disability pension or a rehabilitation measure.

The ‘Ü45-Check’ was conducted as a prospective cross-sectional study to assess the need for prevention and rehabilitation based on preventive health examinations compared to a questionnaire survey. The preventive health examinations were offered to adults aged 45 to 59 years in the federal city of Berlin and the federal state of Brandenburg, Germany. In June 2021, the German Pension Fund (GPF) identified and categorized all individuals insured by them, falling within the age range of 45 to 59 years, and residing in either Berlin or Brandenburg. These individuals were systematically enrolled in the GPF registers and classified into one of three risk groups based on their potential for work disability, as determined by the RI-DP (low risk, moderate risk, and high risk). Participants were randomly selected from each of the three groups and invited by the GPF to participate in the ‘Ü45-Check’ in the following four consecutive calendar years (2021 to 2024). Out of 500,000 people in the register, a total of 9,800 people were invited to the preventive health examinations ‘Ü45-Check’, out of whom 1,241 were interested in participating. A total of 1,040 subjects were finally included in the study (Figure 1). The study design has already been published (17).

### Setting

The ‘Ü45-Check’ was a preventive health examination performed in the outpatient clinic of the Department of Sports Medicine, Charité - Universitätsmedizin Berlin/Humboldt-Universität zu Berlin, Germany. The check-up included a clinical examination and a questionnaire survey. Participants were eligible if they were between 45 and 59 years old, lived in Berlin or Brandenburg, Germany, and were insured with the GPF. Because of the predefined exclusion criteria, individuals with insufficient skills in German or English language, individuals who were already in early retirement, and individuals who have recently had rehabilitation treatment were excluded from the study.

Immediately after the Ü45-Check, the participant received the results of their personal health status based on the examination and discussed their results with the physician.

### Risk index – disability pension

In our research project, the GPF collected and considered the RI-DP during the recruitment process. The RI-DP seemed not to have an impact on whether individuals chose to participate or not in the study. Neither the physicians nor the study participants were provided with any information regarding the specific index values of the subjects; only the project management team had access to this data.

The findings offered by this index can potentially improve the quality of rehabilitation programs as they can be developed with a more targeted approach. The RI-DP, which assesses the risk associated with disability pensions, can be determined by analyzing secondary data from the three years leading up to the reference year. An individual has a low risk if the RI-DP is < 50 points, a moderate risk if the RI-DP is 50 to < 60 points, and a high risk of disability pension if the RI-DP is ≥ 60 points (13, 14).

### Questionnaire (Ü45-screening)

Before the health screening, individuals were asked to complete a web-based questionnaire, which takes approximately 10 min to complete. This two-page questionnaire is an assessment to identify existing prevention and rehabilitation needs. It was developed by another research group as part of the ‘Ü45-Check’ (18, 19) and can be found in the Supplementary Appendix Table S1.

The questionnaire is evaluated by a scoring system where varying values are assigned based on respondents’ answers to each question. Each of the five dimensions (work ability, mental health, functional ability, coping behavior, and sports and exercise behavior) within the questionnaire is assessed separately and weighed differently. The questionnaire had previously been validated and the three dimensions most relevant for the score (work ability, mental health, functional ability) had Cronbach’s Alpha values in the range of 0.79–0.90.

The questionnaire consists of 7 main questions comprising a total of 24 items, distributed across five dimensions as follows: work ability (3 items), mental health (4 items), functional ability (4 items), coping behavior (4 items), sports and exercise behavior (4 items). Participants responded to the questions using different response formats tailored to the content of each dimension. Most items use a Likert-type scale, offering 4 to 5 ordered response options (e.g., “not at all” to “almost every day,” or “without any problems” to “impossible”). One item in the work ability section uses a forced-choice single-response format, and another allows multiple responses to reflect the complexity of the condition. The sports and exercise behavior items use a frequency-based Likert scale ranging from “not at all” to “2 h or more.” These varied response formats were specifically designed to capture the multifaceted nature of each dimension adequately. Three domains are distinguished in the evaluation of the questionnaire: no action needed, prevention program suggested, and rehabilitation program suggested (18, 19).

### Preventive health examination

The preventive health examination lasted a total of approximately 120 min and included anthropometric measures [height, weight, hip, and waist circumference, Bioelectrical Impedance Analysis (BIA)], handgrip strength, twelve-lead resting electrocardiogram (ECG), systolic and diastolic blood pressures, anamnesis, cardiovascular examinations, and blood samples. The detailed presentation of all parameters is published in the study protocol (17). The physical examination mainly focused on the cardiovascular system, the lungs, and the abdomen. Blood analyses were performed under fasting conditions.

In the subsequent consultation with the physician, a focus was paid on the relevant medical history. Medical needs and current health problems were also addressed, and advice for further treatment and evaluation was given. In addition, suggestions on regular exercise, health habits, and a healthy diet were provided.

### Traffic light system

Following the preventive health examination, physicians evaluated the individual’s health using a traffic light system. The assessment was conducted by the physicians in accordance with the dual control principle. Green indicated no additional action was needed, yellow suggested a prevention program, and red indicated a rehabilitation program. These categories represented an increasing health risk, with “green” indicating the lowest risk and “red” indicating the highest or the need for rehabilitation. The study physician evaluated the subject’s health status based on their medical history and examination findings, independent of the Ü45-Screening questionnaire results. Participants received detailed feedback on each measurement and overall health evaluation. Additional examinations were recommended if necessary, with specific guidance tailored to the individual’s risk profile.

### Statistical analysis

Initially, demographic and anthropometric characteristics are described descriptively stratified by gender presenting, mean and standard deviation (sd) for continuous variables and absolute and relative frequencies for categorical variables (cf. Table 1). As none of the variables had a skewed distribution, we refrained from reporting the median and range. The same table, but stratified according to the results of the preventive health examination and the questionnaire, can be found in the supplement S2. To illustrate the differences between the results of the preventive health examination and the questionnaire, a cross table was created, showing the absolute number of overlapping and non-overlapping results and the corresponding percentage of the total of 1,039 cases (cf. Table 2). In addition, a Bowker test was applied, and Cohens’g estimated it (20, 21). Given the exploratory nature of this study, the *p*-values should only be interpreted descriptively.

**Table 1:** Demographic and anthropometric characteristics.

Parameter *N* = 1,040	Total [mean (sd)]	Male [mean (sd)]	Female [mean (sd)]
Gender [*n* (%)]	1,040	631 (61%)	409 (39%)
Age [years]	52.93 (4.17)	52.93 (4.21)	52.93 (4.12)
Body-Mass-Index (BMI) [kg/m^2^]	26.89 (4.90)	27.45 (4.36)	26.02 (5.51)
Waist-to-Hip-Ratio (WHR)	0.90 (0.09)	0.95 (0.07)	0.82 (0.07)
Percent Body Fat (PBF) (*n* = 1,024) [%]	30.19 (9.11)	27.810 (7.90)	33.84 (9.61)
Visceral Fat Area (VFA) (*n* = 1,024) [cm^2^]	118.30 (53.44)	110.97 (47.61)	129.46 (59.61)
Skeletal Muscle Mass (SMM) (*n* = 1,024) [kg]	31.28 (7.02)	35.69 (5.00)	24.55 (3.34)
Employment status [*n* (%)]			
Employed	936 (90%)	560 (89%)	376 (92%)
Currently not employed	73 (7%)	48 (7.6%)	25 (6.1%)
Sick leave	20 (1.9%)	14 (2.2%)	6 (1.5%)
NA	11 (1.1%)	9 (1.4%)	2 (0.5%)

BMI, body-mass-index; PBF, percent body fat; sd, standard deviation; SMM, skeletal muscle mass; VFA, visceral fat area; WHR, waist-to-hip-ratio; NA, no answer (information not provided by study participant).

**Table 2:** Crosstable describing preventive health examinations (by physicians) x questionnaire survey (Ü45 questionnaire).

Preventive health examinations	Questionnaire survey	Preventive health examinations			
		No additional action needed	Prevention program suggested	Rehabilitation program suggested	Total
Questionnaire survey	No additional action needed	123 (12%)	553 (53%)	57 (5%)	733 (70%)
	Prevention program suggested	16 (2%)	127 (12%)	72 (7%)	215 (21%)
	Rehabilitation program suggested	2 (0%)	37 (4%)	52 (5%)	91 (9%)
	Total	141 (14%)	717 (69%)	181 (17%)	1,039 (100%)

Line, questionnaire survey; column, preventive health examinations. Displayed are the absolute numbers and percentages in relation to the total sample (*n* = 1,039).

**Figure 2:**
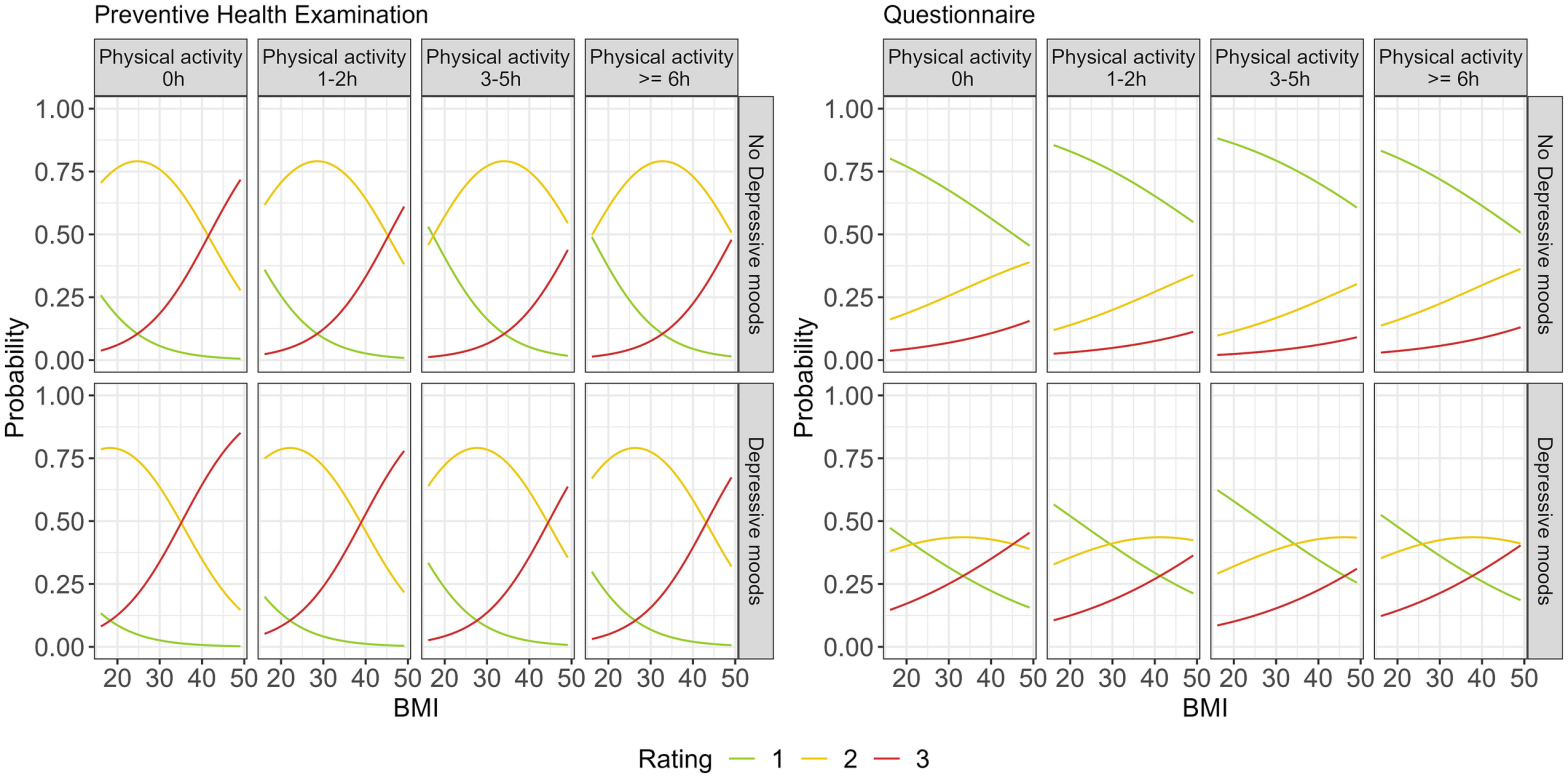
Predicted probability of needing prevention or rehabilitation in relation to hours of physical activity, depressive moods, and BMI based on the preventive health examination and the Ü45-Screening questionnaire; 1 = no additional action needed, 2 = prevention program suggested, 3 = rehabilitation program suggested.

**Figure 3:**
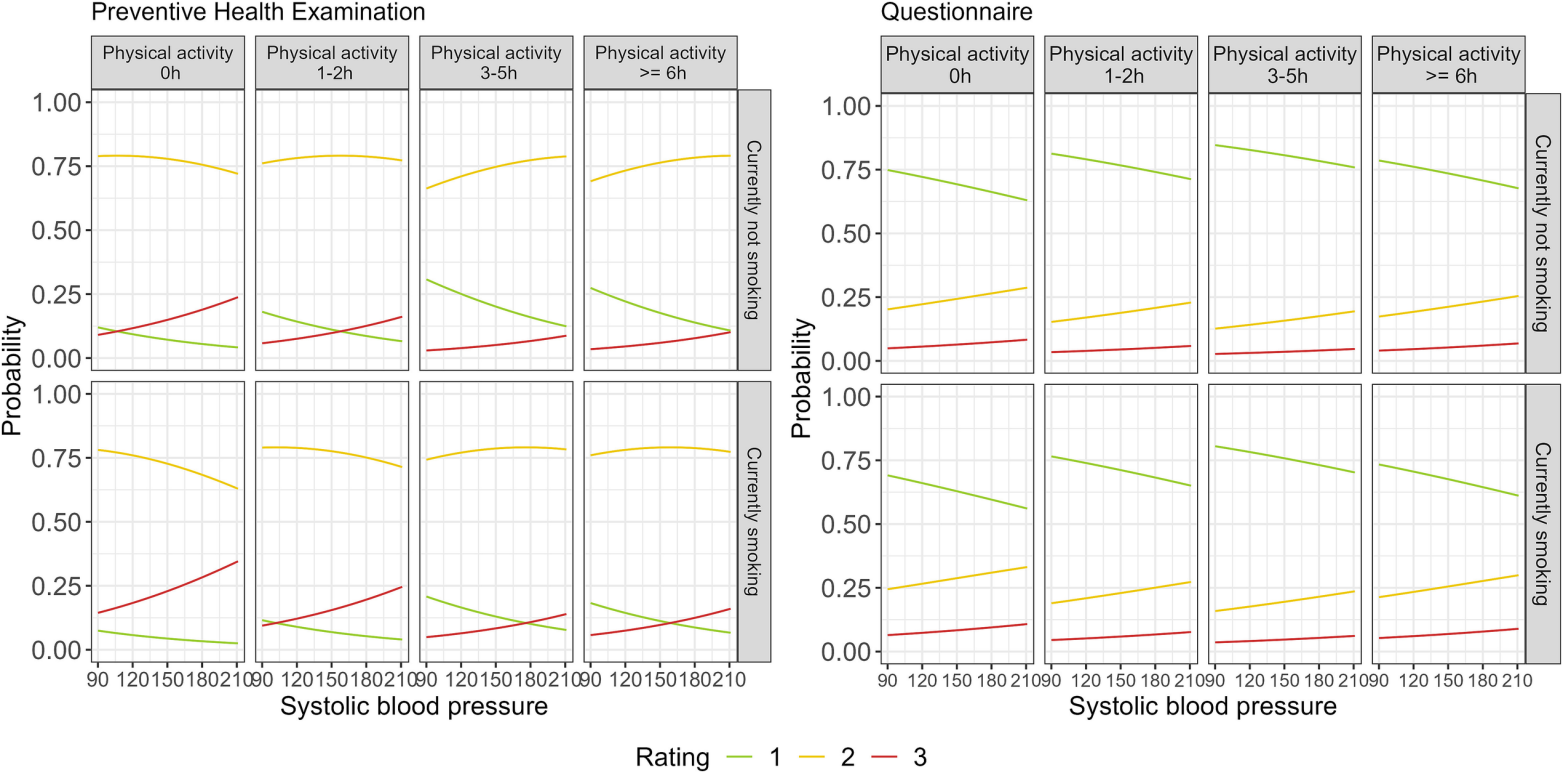
Predicted probability of needing prevention or rehabilitation in relation to hours of physical activity, currently smoking, and systolic blood pressure based on the preventive health examination and the Ü45-Screening questionnaire; 1 = no additional action needed, 2 = prevention program suggested, 3 = rehabilitation program suggested.

We also asked the doctors involved in the study which measurements they particularly consider when making their decision. To analyze the association of these variables on the final decision taking all levels of the outcome into account, we estimated a multivariable cumulative ordinal regression model. As this is not a confirmatory study, the model falls within the scope of descriptive models. Multivariable models were estimated in order to capture the entire dependency structure of the variables, and univariable models were refrained from. Thereby, the variables Body-Mass-Index (BMI) [kg/m^2^], Waist-to-Hip-Ratio (WHR) [per 0.1 cm/cm], gender [female/male], Systolic Blood Pressure (SBP) [per 10 mmHg], Low-density Lipoprotein (LDL) cholesterol [per 10 mg/dL], HbA1c [%], hand grip strength [kg], currently smoking [yes/no], depressive moods [yes/no] and physical activity [0 h/1-2 h/3-5 h/ ≥ 6 h] were analyzed. All variables were continuous, except the binary variable gender and the categorical variable physical activity. The same variables were also used to analyze whether these variables also had an indirect influence on the results of the questionnaire in a second multivariable cumulative regression model. All continuous variables included in the model had a Pearson correlation of lower than 0.5 (Figure S3). Graphical inspections of the variables did not show any outliers. Since only 33 (3.2%) of the data was missing, a complete case analysis was done. Results are reported as odds ratios (OR) and their corresponding 95%-confidence intervals (CI). Furthermore, the predictions for differing BMI, depressive moods, and physical activity and the predictions for differing SBP, current smoking, and physical activity for both models are visualized in Figures 2, 3, respectively. For this illustration, all other variables are set to the mean value of the population if it is a continuous variable or to the most frequent category if it is a categorical variable. For a more detailed explanation of the plots, we refer to the results section.

Similarities between the RI-DP and the questionnaire were also analyzed using a cross table (Table 3).

**Table 3:** Comparison of assessment results with the risk index-disability pension.

	*N* = 1,040	Low risk *n* = 540 (%)	Moderate risk *n* = 307 (%)	High risk *n* = 193 (%)
Questionnaire survey	no additional action needed	425 (79%)	207 (67%)	101 (52%)
	prevention program suggested	87 (16%)	69 (22%)	60 (31%)
	rehabilitation program suggested	28 (5.2%)	31 (10%)	32 (17%)
Preventive health examinations	no additional action needed	93 (17%)	37 (12%)	11 (5.7%)
	prevention program suggested	387 (72%)	218 (71%)	112 (58%)
	rehabilitation program suggested	60 (11%)	52 (17%)	69 (36%)
	No answer (NA)	0 (0%)	0 (0%)	1 (0.5%)

Line, questionnaire survey and preventive health examinations; column, risk index-disability pension (RI-DP); NA, no answer (information not provided by study participant).

For the analysis and figures, the software R (version: 4.3.1) was used, and for the ordinal regression, the function polr() (R-package MASS version: 7.3) was used.

### Ethics approval and consent to participate

Ethics approval was obtained by the “Ethics Committee of the Faculty of Culture, Social and Educational Sciences, Humboldt-Universität zu Berlin” on August 20, 2020 (reference number: HU-KSBF-EK_2020_0010). The work described was performed in accordance with the Declaration of Helsinki for experiments on humans. All subjects were informed by the study staff about the study procedure, subsequent data storage, and confidentiality and pseudonymity of the data. Written informed consent was obtained from all subjects for the study center to use the data for research analyses and publication of the data.

## Results

1,040 individuals [631 men (61%) and 409 women (39%)] with a mean age of 52.93 years (sd = 4.17) participated in the study. The data on body composition show a mean BMI of 26.89 kg/m^2^ (sd = 4.90) and a mean Percent Body Fat (PBF) of 30.19% (sd = 9.11). The mean value of the Visceral Fat Area (VFA) of the participants is 118.30 cm^2^ (sd = 53.44) above the validated threshold of 100 cm^2^. The data underline gender differences in anthropometric characteristics such as BMI, WHR, PBF, VFA, and Skeletal Muscle Mass (SMM). Men tend to have higher values for BMI, WHR, and SMM, while women exhibit higher values for PBF and VFA (cf. Table 1). According to the World Health Organization (WHO) recommendations, the BMI of the participants was distributed as follows: 8 (0.8%) underweight (BMI < 18.5 kg/m^2^), 407 (39.1%) normal weight (18.5–24.9 kg/m^2^), 370 (35.6%) pre-obese (25–29.9 kg/m^2^), 197 (18.9%) obese class I (30–34.9 kg/m^2^), 45 (4.3%) obese class II (35–39.9 kg/m^2^) and 13 (1.3%) obese class III (≥ 40 kg/m^2^). The mean BMI was 26.89 kg/m^2^ (sd = 4.90) for all, 27.45 kg/m^2^ (sd = 4.36) for men and 26.02 kg/m^2^ (sd = 5.51) for women. The mean values show that many participants, especially men, are overweight, according to the WHO (22).

For WHR, men have a mean value of 0.95 (sd = 0.07), while women have a mean value of 0.82 (sd = 0.07). Similarly, a WHR above 0.90 for men and 0.85 for women is indicative of a higher risk of cardiovascular disease, which aligns with the higher WHR values observed in men in this study. In terms of BPF, men have a mean PBF of 27.80% (sd = 7.90), whereas women have a higher mean PBF of 33.84% (sd = 9.61). The differences in PBF also underscore the distinct physiological and metabolic profiles between men and women, with women naturally having higher PBF (23). Reference values (age group 40–59 years) for average healthy PBF ranges are approximately 18.5–24.9% for men and 19.5–30% for women (24), indicating that many participants, particularly women, might exceed these healthy ranges. The VFA also shows expected differences, with men having a mean VFA of 110.97 cm^2^ (sd = 47.61) and women having a higher mean VFA of 129.46 cm^2^ (sd = 59.61). The VFA data also suggests that a substantial number of participants, especially women, are above the 100 cm^2^ threshold associated with increased metabolic risk. Lastly, men exhibit a higher SMM with a mean of 35.69 kg (sd = 5.00) compared to women, who have a mean SMM of 24.55 kg (sd = 3.34).

### Prevention or rehabilitation needs

The questionnaire, as well as the physicians during the preventive health examination, determine the prevention or rehabilitation needs of the participants independently of each other. According to the questionnaire survey, the prevention or rehabilitation needs of the 1,039 participants are as follows: no additional action needed (green) *n* = 733 (70%), prevention program suggested (yellow) *n* = 215 (21%), rehabilitation program suggested (red) *n* = 91 (9%). As part of the preventive health examination, the physicians assessed the prevention or rehabilitation needs of the 1,039 participants as follows: no additional action needed (green) *n* = 141 (14%), prevention program suggested (yellow) *n* = 717 (69%), rehabilitation program suggested (red) *n* = 181 (17%). Table 2 displays the absolute and relative frequencies of the variables between the two assessments. The table shows that the physicians’ assessments differ from the results of the questionnaire. It is noteworthy that the results of the questionnaire and the clinical examination matched only 29% green, *n* = 123 (12%), yellow, *n* = 127 (12%), and red, *n* = 52 (5%). The Bowker test indicated an asymmetry and, thereby, differences between the questionnaire and preventive health examination (*p*-value = 2.2 * 10–16, Cohen’s g = 0.425). Due to the large sample size, the test results should be interpreted with caution, and more attention should be paid to the interpretation of the effects.

In particular, physicians often suggested a prevention program whereas, the questionnaire suggested that no additional measures were needed [*n* = 553 (53%)]. According to the questionnaire, the majority of the participants did not need an intervention. In contrast to this, the physicians found, based on clinical parameters, that at least a preventive action should be taken. This means that the content of the questionnaire and the self-assessment differ from the physicians’ assessment. The overlap in the rehabilitation program category suggests that both assessments consistently identify individuals who need a higher level of intervention.

Supplementary Appendix Table S2 presents a stratified descriptive analysis regarding demographic and anthropometric characteristics of the 1,039 individuals based on the results of preventive health examinations (green, *n* = 141; yellow, *n* = 717; red, *n* = 181) and the results from the questionnaire (green, *n* = 733; yellow, *n* = 216; red, *n* = 91).

The gender distribution across the groups is relatively balanced, with a slight predominance of men in all groups, particularly in the “yellow” group. Age increases slightly from the “green” to the “red” group, suggesting that older individuals may have higher rehabilitation needs. BMI, PBF, and VFA increase from the “green” to the “red” group, indicating a higher prevalence of overweight and obesity among individuals with greater rehabilitation needs. Both screening tools indicate similar trends across the groups: an increase in age, BMI, PBF, and VFA, with a decline in employment status from the “green” to the “red” group. These trends suggest that a greater need for rehabilitation is associated with older age, higher obesity rates, and unemployment.

### Multivariable cumulative ordinal regression models to evaluate the association of parameters on prevention and rehabilitation needs

Multivariable cumulative ordinal regression models were used to identify potential predictors from the preventive health examination and questionnaire to determine the need for prevention and rehabilitation. The parameters examined included BMI, WHR, gender, SBP, LDL cholesterol, HbA1c, handgrip strength, smoking status, depressive mood, and physical activity.

Table 4 presents the OR and their 95%-CI for all predictors. The multivariable cumulative ordinal regression models show that individual factors may influence prevention or rehabilitation needs within the traffic light system based on the preventive health examination and the questionnaire.

**Table 4:** Results of the multivariable cumulative ordinal regression models assessing potential risk factors for prevention or rehabilitation needs within the traffic light system based on the preventive health examination and the questionnaire.

Parameter *N* = 1,007	Preventive health examination	Questionnaire
	Odds Ratio (OR) [95%-CI]	
Body-mass-index (BMI) [per 1 kg/cm^2^]	1.135 [1.094; 1.178]	1.049 [1.012; 1.087]
Waist-to-hip-ratio (WHR) [per 0.1 cm/cm]	1.048 [1.022; 1.075]	1.036 [1.009; 1.063]
Gender		
Female	Reference	Reference
Male	0.679 [0.406; 1.131]	1.790 [1.057; 3.046]
Systolic Blood Pressure (SBP) [per 10 mmHg]	1.099 [1.009; 1.197]	1.047 [0.960; 1.142]
LDL cholesterol [per 10 mg/dl]	0.937 [0.900; 0.975]	0.924 [0.885; 0.964]
HbA1c [%]	0.926 [0.803; 1.043]	0.923 [0.731; 1.075]
Hand grip strength [per 1 kg]	0.975 [0.959; 0.990]	0.931 [0.915; 0.948]
Currently Smoking	1.691 [1.212; 2.366]	1.331 [0.947; 1.862]
Depressive moods	2.254 [1.565; 3.254]	4.506 [3.216; 6.322]
Physical activity		
0 h	Reference	Reference
1–2 h	0.618 [0.436; 0.874]	0.686 [0.481; 0.977]
3–5 h	0.307 [0.204; 0.460]	0.541 [0.353; 0.822]
≥ 6 h	0.361 [0.218; 0.598]	0.812 [0.477; 1.358]

CI, confidence interval; BMI, body-mass-index; OR, odds ratio; SBP, systolic blood pressure; WHR, waist-to-hip-ratio.

In summary, both the preventive health examination and the questionnaire identified several potential predictors influencing the chance of falling into a higher category. BMI, LDL cholesterol, handgrip strength, depressive mood, and certain levels of physical activity showed similar associations in both assessments. Specifically, an increase in BMI was associated with higher odds of being in a higher category in both the preventive health examination [OR: 1.135 95%-CI (1.094; 1.178)] and the questionnaire [OR: 1.012 (1.087)], although the effect size was smaller for the questionnaire. LDL cholesterol showed a decrease in odds for a higher category with increasing levels (10 mg/dL) in both assessments [preventive health examination OR: 0.937 (0.900; 0.975); questionnaire OR: 0.924 (0.885; 0.964)]. Depressive mood strongly increased the odds of being in a higher category in both assessments, with a stronger association in the questionnaire [preventive health examination OR: 2.254 (1.563; 3.254); questionnaire OR: 4.506 (3.216; 6.322)]. Increased physical activity was associated with lower odds in both assessments, although the effect sizes varied [preventive health examination OR for 3–5 h/week: 0.307 (0.204; 0.460) and ≥6 h/week: 0.361 (0.218; 0.598); questionnaire OR for 3–5 h/week: 0.541 (0.353; 0.822) and ≥6 h/week: 0.812 (0.477; 1.358)] compared to no physical activity. All results are to be understood descriptively and do not imply a causal relation.

However, there were variables that showed different results between the two assessments. Gender had contrasting ORs, with men having lower odds of being in a higher category than females in the preventive health examination [OR: 0.679 (0.406; 1.131)] but higher odds in the questionnaire [OR: 1.790 (1.057; 3.046)] than women. Current smoking status increased the odds in the preventive health examination [OR: 1.691 (1.212; 2.366)] but had a smaller association in the questionnaire [OR: 1.331 (0.947; 1.862)].

In summary, in the preventive health examination, predictors influencing the chance of falling into a higher category included BMI, WHR, SBP, LDL cholesterol, handgrip strength, current smoking status, depressive mood, and physical activity. Gender and HbA1c levels showed no association.

Additionally, predictors influencing a higher category in the questionnaire included BMI, WHR, gender, LDL cholesterol, handgrip strength, depressed moods, and a certain level of physical activity. Blood pressure, HbA1c level, and current smoking status showed no association in our models.

Figure 2 displays prediction plots illustrating the probability of individuals falling into one of three categories based on BMI, depressive moods, and physical activity: (1) no action needed, (2) prevention program suggested, and (3) rehabilitation program suggested. Recall that we are in the setting of an ordinal regression model. Hence, certain standard measures, such as marginal effects, cannot be applied in the same way as in standard logistic regression models. Therefore, we chose to use a graphical display of the predicted probabilities. This should address a similar perspective and should ease interpretation in the setting of ordinal regression modeling.

For “green” (no action needed), both graphs show higher probabilities for individuals with lower BMI, no depressive moods, and higher levels of physical activity. An example shows that a person who does not do physical activities has no depressive moods, and has a BMI of 20 would most likely end up in “yellow” according to the preventive health examination and in “green” according to the Questionnaire.

The preventive health examination offers clearer and more distinct trends (e.g., overlap of the “yellow” and “red” curves with increasing BMI and depressive moods), whereas the questionnaire exhibits greater variability and less distinct patterns. The figure indicates that the probability of falling into the “yellow” category, according to the questionnaire, is relatively constant, regardless of the level of BMI. However, it is noteworthy that depressive moods exert a greater association with the questionnaire results, followed by a notable decline in the probability of falling into the “green” category. For “red” (rehabilitation program suggested), both diagrams indicate the highest probabilities for individuals with higher BMI, depressive moods, and lower levels of physical activity.

These prediction plots highlight the varying probability of required interventions (prevention or rehabilitation needs) based on the assessed health parameter. These similarities and differences underline the complementary nature of both approaches. While preventive health examinations provide detailed health assessments, questionnaires offer a practical method for initial screening and risk stratification.

Figure 3 displays prediction plots illustrating the probability of individuals falling into one of three categories based on blood pressure (BP), smoking status, and physical activity, while all other variables remain constant.

In the context of a preventive health examination, the probability of requiring rehabilitation services is elevated among participants who engage in tobacco use and present with elevated BP. In contrast, both BP and smoking show a minimal relation to the questionnaire results. The number of hours spent engaging in physical activity also exerts only a slight association with the probability of falling into a higher category on the questionnaire. In the preventive health examination, a more pronounced trend emerges, indicating that the probability of requiring rehabilitation is highest among individuals who engage in no physical activity and have high BP compared to those who engage in other levels of physical activity.

This indicates that the questionnaire is inadequate for assessing the parameters of physical activity, smoking, and BP, given that these factors are only addressed indirectly.

### Risk index – disability pension in relation to prevention or rehabilitation needs

The RI-DP is divided into three levels, depending on the level of risk of early retirement [low risk *n* = 540 (52%), moderate risk *n* = 307 (30%), and high risk *n* = 193 (19%)]. Table 3 compares the results of the questionnaire survey with those from preventive health examinations concerning the RI-DP and recommended measures for three different risk categories: low risk, moderate risk, and high risk.

The results show that *n* = 425 (79%) with a low RI-DP, according to the questionnaire survey, do not require any additional action compared to the results of the preventive health examinations [*n* = 93 (17%)]. In contrast, people with high RI-DP are more likely to need a rehabilitation program according to preventive health examinations *n* = 69 (36%) than according to the questionnaire survey *n* = 32 (17%). This indicates that a greater need for intervention was recognized in the sample during the preventive health examinations than in the questionnaire surveys, irrespective of the RI-DP.

## Discussion

The results of the preventive health examination differ from the results of the questionnaire survey. Both tools are designed to identify a need for prevention or rehabilitation. The study findings revealed a notable discrepancy between individuals initially classified as requiring no additional action based on questionnaire assessments and those recommended prevention programs in the preventive health examination (*n* = 553). This discrepancy underlines the value of integrating questionnaire surveys with preventive health examinations. Compared to the questionnaire, the preventive health examination can reveal additional health needs that the questionnaire alone does not (25, 26). The analysis of the multivariable regression models highlights that. However, due to the descriptive character of the study, all results are to be interpreted descriptively and do not imply a causal relation. While several potential predictors overlap between the preventive health examination and the questionnaire, their association varies. This is reflected in the fact that parameters like BMI, WHR, LDL cholesterol, handgrip strength, depressive moods, and physical activity showed a potential association on falling into a higher category (no action needed, prevention program suggested, rehabilitation program suggested). In contrast, SBP and smoking status only seemed to be associated with the higher classification in the preventive health examination and gender only in the questionnaire. As a result, the differences in the specific parameters that lead to a higher score indicate the importance of taking context more than isolated factors into account when assessing health.

The results of this study show that it might be important to combine questionnaire surveys with preventive health checks to reach a well-founded health evaluation. The questionnaire survey was found to underestimate the need for prevention (21%) among over 45-year-olds compared with the medical examination (69%), which is why it makes sense to use both tools together. This is further supported by the results shown in Figure 2. Here, the health examination and the questionnaire are compared in their classification of the need for prevention or rehabilitation with regard to the parameters of physical activity in hours, BMI, and depressive moods. As already described in the results, a person who does not exercise, does not have depressive moods, and has a BMI of 20 would be classified as “green” by the questionnaire and as “yellow” by the medical examination. If the questionnaire were used alone, it would substantially underestimate the need for prevention, as the results of this study show. However, as the WHO states, a lack of exercise is a major health risk factor that can lead to numerous diseases (27–29). Even a low BMI does not necessarily mean that a person does not have increased visceral fat or sufficient skeletal muscle mass, which are predictors for minimizing the risk of diseases (e.g., frailty syndrome or osteoporosis) and is not recorded by the BMI (30–32). This is underlined by the fact that the questionnaire cannot adequately assess the parameters of physical activity, smoking status, and blood pressure (Figure 3). Nevertheless, the questionnaire should not be disregarded when assessing the need for prevention or rehabilitation. It was shown that the depressive mood parameter had a greater association than in the preventive health examination. Consequently, the questionnaire is better at capturing depressive moods than the preventive health examination (Table 4). Given the increase in mental illness in Germany and the associated risks of illness and incapacity to work, the adequate detection of this parameter is an important factor in assessing the need for prevention or rehabilitation (33–35). Against this background, the use of the Ü45 questionnaire can be justified. This example shows that a combination of screening tools can increase the accuracy and scope of health assessments, as parameters can be recorded differently by different tools (36, 37).

The validity and reliability of the questionnaire have been confirmed in a large-scale study with a high number of participants, where the relevant dimensions contributing to the score demonstrated high internal consistency (Cronbach’s Alpha: 0.79–0.90). This underscores the robustness of the questionnaire as a screening instrument. The detailed validation process, including the reliability assessment, is documented in the final German study reports, which have been cited accordingly (18, 19). Given these findings, combining both tools - considering their respective strengths - can enhance the accuracy and scope of preventive health assessments.

The integration of these screenings into routine clinical practice could, therefore, promise timely and tailored preventive interventions for a wider population, thereby reducing the incidence of more serious health outcomes (38–42). The feasibility of this integration depends on the ability of the healthcare system to support regular and efficient implementation. In 2014, for example, the GPF spent around six billion euros on medical rehabilitation and benefits for participation in working life and social security contributions (43). In the same year, the healthcare system paid around two point six billion euros for rehabilitation services. Effective and helpful rehabilitation should not be seen as a financial burden by any sector. It is desirable to offer a treatment plan for a patient based on a valid, objective, and reliable screening system that aims to identify the individual’s risk factors, needs, and opportunities for involvement (39, 43, 44). A well-researched preventive health screening program should have a positive impact on the health care system and government costs, which is also becoming a focus at the international level (45, 46). While organizations like the American Cancer Society, American Diabetes Association, and American Heart Association emphasize the importance of preventive measures in reducing the burden of major diseases such as cardiovascular disease, cancer, and diabetes, the U. S. Preventive Services Task Force and Swiss Medical Board provide updated, evidence-based recommendations on screening practices (47, 48). Furthermore, a structured preventive health strategy can substantially bolster patient compliance. Comprehensive patient education on health status and the rationale underlying recommended interventions promote enhanced adherence to preventive measures (39). This holistic approach elicits a sense of accountability and proactive engagement in health management, thereby promoting improved health outcomes.

Based on the data from this study, the use of a preventive health screening consisting of a preventive health examination and a questionnaire survey can be recommended. This could be limited to the most relevant parameters, considering a cost–benefit analysis, and thus serve the economic demands of the political actors as well as the medical demands of the actors in the healthcare industry. In an optimized preventive examination (e.g., a reduced version of the Ü45-Check with consideration of costs), some parameters that have not demonstrated any associations could be omitted. It should be emphasized that some individuals exhibited abnormalities, but this may be cost-prohibitive for the implementation of a standardized national examination. In light of the findings, it can be concluded that the performance of the electrocardiogram (ECG) was not a relevant factor, as minimal abnormalities were identified in the sample. Additionally, the laboratory tests could also be discussed, as the blood values are a snapshot. In both tools, the HbA1c level seemed to be associated with the classification into a higher category (no action required, prevention program suggested, rehabilitation program suggested) and would, according to this study, be negligible. However, HbA1c has been shown to be an important indicator for the diagnosis and prognosis of diabetes mellitus. It can be used for early detection of Type 2 diabetes and thus remains to be discussed (49, 50). On the other hand, high BMI, physical inactivity, and depressive mood had the greatest association on classification in the red category (rehabilitation program recommended) in both tools and should be in greater focus from the point of view of prevention.

In this study, the individual RI-DP of the test subjects was considered and also highlighted in the results. In comparison with a prediction index from routine data [such as the RI-DP (13)] for conventional screening and health examinations, it becomes clear that an index cannot replace a preventive examination. This is supported by Table 3, which shows that there was partially no agreement between the assessment of the RI-DP and the questionnaire or the preventive health examination. Despite potential upfront costs, these supplementary examinations hold promise for yielding long-term savings by averting more severe and costly health complications. Within the context of Germany’s healthcare framework, which prioritizes preventive care, the benefits of these comprehensive examinations may outweigh the associated costs, thus presenting a favorable cost–benefit proposition. Nevertheless, an index created from routine data is a valuable addition to the existing toolkit (51, 52). However, in view of the fact that regulations, implementation, preventive services, and preventive health examinations are currently rather unclear, a combined analysis of the three evaluations can promise a well-founded medical assessment. The parameters that differentiate the RI-DP from the questionnaire remain to be discussed, as well as the causes of the discrepancies in the health assessment. However, this can be an important factor in establishing a well-founded health screening in Germany, as it promotes an improved cost–benefit profile.

Optimal implementation of preventive health examinations depends on a regular assessment of the population-specific needs and risk profiles (53–55). While annual or biennial check-ups may suffice for the general population, high-risk cohorts may benefit from more frequent assessments. Seamless integration into routine practice mandates streamlined processes, potentially utilizing digital health infrastructure and automated reminders to ensure adherence. Comprehensive training of healthcare practitioners in conducting these examinations and interpreting results is paramount for effective integration (56).

In addition to routine check-ups, several supplementary strategies can enhance preventive care. Health education campaigns play a crucial role in disseminating knowledge about preventive care and promoting healthy lifestyle practices (57–59). Digital health tools offer a promising option for health monitoring and delivering personalized guidance to individuals. Community health initiatives, including local programs focused on promoting physical activity, ensuring nutritional adequacy, and facilitating regular health assessments, contribute substantially to preventive care efforts. Furthermore, workplace health initiatives, which encourage employers to provide preventive health screenings, are vital for fostering a healthy workforce and promoting preventive health measures (60–62).

### Strengths

One of the primary strengths of the study is the large sample size with a very small amount of missing data, which enhances the reliability and generalizability of the findings. With 1,040 participants, the study is well-powered to detect associations and provides a robust dataset for analysis. Additionally, the early detection of risk factors is crucial for achieving better health outcomes and potentially preventing the onset of associated diseases or secondary complications (40, 41, 63). This proactive approach may not only help reduce the financial burden on the healthcare system but also indicate that comprehensive check-ups can offer significant health benefits to individuals (42).

The specific examinations within the Ü45-Check have been carefully selected to provide meaningful results and to ensure their economic feasibility for use in various settings, such as primary care practices (17). Notable strengths include the use of standardized methods for assessing body composition and the application of established measurement techniques, such as ECG, and handgrip strength diagnostics (64). The study also highlights the importance of combining questionnaire surveys with preventive health examinations to ensure a comprehensive assessment of an individual’s health needs. This integrated approach might increase the effectiveness of health interventions by ensuring that individuals receive appropriate recommendations based on a combination of self-reported data and clinical assessments.

Further strengths of the study include the use of diverse assessment tools, which provide a comprehensive evaluation of health by capturing both objective clinical data and subjective self-reported information. The standardized protocols and measurement techniques ensure consistency and accuracy in data collection, enhancing the validity of the results. By focusing on individuals aged 45 to 59 years, a critical age group for the onset of many chronic conditions, the study allows for early intervention and timely preventive measures.

Moreover, the health status evaluations by physicians were conducted independently of the questionnaire results, reducing bias and providing an objective assessment of health needs. These assessments were further strengthened by the involvement of several specialists, particularly in internal medicine and cardiology, to ensure that assessments were made from different expert perspectives. In addition, the implementation of the “four-eye principle,” whereby a chief physician reviewed each assessment, added an extra layer of scrutiny and reliability to the assessments. Participants received detailed feedback on their health status and personalized recommendations, which could encourage proactive health management and improve individual health outcomes.

### Limitations

Self-selection is closely related to health consciousness, which represents a state of heightened awareness and voluntary engagement in health-promoting activities, behaviors, and lifestyle choices. A potential source of bias lies in the fact that individuals who feel unwell may be more inclined to accept the invitation to participate in the Ü45-Check. On the other hand, individuals with a higher risk profile may choose not to participate in preventive medical check-ups. The RI-DP could serve as an active strategy to encourage greater participation in these check-ups.

The study design was cross-sectional, which means that we cannot definitively determine whether participants followed the recommendations for prevention or rehabilitation. There is also the question of which tool determines the real need, which we cannot answer with our cross-sectional survey because we cannot look into the participants’ future. However, it is important to note that follow-up is both planned and requested as part of the informed consent process. Furthermore, no causal effects can be estimated within this study.

## Conclusion

The results of the Ü45-Check program, which was commissioned by the German government, have provided an important scientific basis that holds importance for primary health care.

Within this study, the data show that it might be of importance to combine questionnaire surveys with preventive health examinations to ensure a comprehensive assessment of an individual’s health needs. The preventive health examinations appear to provide a more thorough assessment, often suggesting preventive or rehabilitative measures that the questionnaire alone may not capture. This integrated approach might increase the effectiveness of health interventions by ensuring that individuals receive appropriate recommendations based on a combination of self-reported data and clinical assessments. Effective screening programs thrive on the collaboration of various organizations and professionals, both within and outside the healthcare sector, to ensure their success (65).

## Data Availability

The raw data supporting the conclusions of this article will be made available by the authors, without undue reservation.
