# Supplementary material for: Evaluation of a validated questionnaire to assess the need for prevention or rehabilitation by preventive health examinations: a cross-sectional study of German employees aged 45 to 59 years (Ü45-check)
Source: Front Public Health. 2025 Jul 16;13:1480312. doi: 10.3389/fpubh.2025.1480312 (PMC12307417; doi:10.3389/fpubh.2025.1480312)
Supplement: Supplementary file 1 [file Supplementary_file_1.pdf]

Work Ability

1. How long were you on sick leave in the past 12 months?

☐ not at all

☐ 1 day to 6 weeks

☐ more than 6 weeks up to 3 months

☐ more than 3 months up to 6 months

☐ more than 6 months

2. Does any illness or impairment currently interfere with your work?
Please tick everything that applies to you. Multiple answers are possible. If you are currently unemployed, refer to your last performed job.

☐ I have no impairment / I have no illness

☐ I can perform my work, but I have complaints

☐ I'm sometimes forced to work slower or to change my working methods

☐ I'm often forced to work slower or to change my working methods

☐ Because of my illness, I'm only able to work part-time

☐ I can no longer work at all

3. If you think of your current health status and your occupational performance: how do you envision your professional future?
Please tick the most applicable option only.

I think that in the next 5 years I'll probably…

☐ …have no severe health impairments that will hinder me from performing at my work place

☐ … be able to continue my profession, but I will have to change my work place due to health impairments

☐ … not be able to continue my profession due to health impairments

☐ … not be able to work at all

Mental Health

4. In the last 4 weeks, how often did you feel impaired by the following complaints?
Please put a cross in each line.

	not at all	on single days	on more than half of the days	almost every day
Little interest or joy in your activities	☐	☐	☐	☐
Dejection, melancholy, hopelessness	☐	☐	☐	☐
Nervousness, anxiousness, tension	☐	☐	☐	☐
Not being able to stop or control worries	☐	☐	☐	☐

Functional Ability

5. In the last 4 weeks, to what degree were you able to perform the following?
Please put a cross in each line.

	without any problems	little difficulties	great difficulties	impossible
Carry a heavy object over 5 meters	☐	☐	☐	☐
Perform physical work for 30 minutes	☐	☐	☐	☐
Stand for 30 minutes without a break	☐	☐	☐	☐
Climbing a staircase over 3 floors	☐	☐	☐	☐

Coping Behavior

6. In the last 4 weeks, how well were you able to cope with the following stress situations? *Please put a cross in each line.*

	very good	better	worse	not at all
Time and appointment pressure	☐	☐	☐	☐
Conflicts, emotionally stressful situations	☐	☐	☐	☐
High responsibility, high demands	☐	☐	☐	☐
Physical load	☐	☐	☐	☐

Sports and Exercise Behavior

7. In the last 4 weeks, how many hours did you perform the following physical activities? *Please put a cross in each line.*

	2 hours or more	1 hour up to 2 hours	up to 1 hour	not at all
Physical exercise	☐	☐	☐	☐
Cycling or running	☐	☐	☐	☐
Physical activity at work	☐	☐	☐	☐
Activities at home	☐	☐	☐	☐
