# Supplementary material for: Evaluation of a validated questionnaire to assess the need for prevention or rehabilitation by preventive health examinations: a cross-sectional study of German employees aged 45 to 59 years (Ü45-check)
Source: Front Public Health. 2025 Jul 16;13:1480312. doi: 10.3389/fpubh.2025.1480312 (PMC12307417; doi:10.3389/fpubh.2025.1480312)
Supplement: Supplementary file 2 [file Table_1.docx]

**Appendix**

**Table S1** Demographic and anthropometric characteristics stratified according to preventive health examination and questionnaire survey

|  | preventive health examination | | | questionnaire survey | | |
| --- | --- | --- | --- | --- | --- | --- |
| Parameter  N = 1,039 | green,  n = 141  (mean (sd)) | yellow,  n = 717  (mean (sd)) | red,  n = 181  (mean (sd)) | green,  n = 733  (mean (sd)) | yellow,  n = 216  (mean (sd)) | red,  n = 91  (mean (sd)) |
| Gender (n (%)) |  |  |  |  |  |  |
| *male* | 80 (57%) | 450 (63%) | 100 (55%) | 460 (63%) | 119 (55%) | 52 (57%) |
| *female* | 61 (43%) | 267 (37%) | 81 (45%) | 273 (37%) | 97 (45%) | 39 (43%) |
| Age | 52.28 (4.35) | 52.85 (4.24) | 53.78 (3.58) | 52.68 (4.24) | 53.72 (3.98) | 53.14 (3.85) |
| Body-Mass-Index (BMI) [kg/m²] | 23.20 (2.74) | 26.95 (4.41) | 29.53 (6.14) | 26.31 (4.40) | 27.88 (5.43) | 29.18 (6.22) |
| Percent Body Fat (PBF) (n = 1,024) [%] | 26.07 (8.18) | 30.14 (8.74) | 33.63 (9.89) | 29.34 (8.71) | 32.82 (9.51) | 33.22 (10.20) |
| Visceral Fat Area (VFA) (n = 1,024) [cm²] | 75.64 (27.68) | 119.02 (48.84) | 148.86 (63.52) | 110.70 (48.57) | 134.03 (59.09) | 142.63 (61.72) |
| Skeletal Muscle Mass (SMM) (n = 1,024) [kg] | 30.58 (6.50) | 31.50 (7.07) | 30.91 (7.18) | 31.67 (7.06) | 30.59 (6.91) | 29.70 (6.66) |
| Employment status (n (%)) |  |  |  |  |  |  |
| *employed* | 138 (98%) | 659 (92%) | 138 (76%) | 703 (96%) | 183 (85%) | 50 (55%) |
| *currently not employed*  *sick leave* | 3 (2.1%)  0 (0%) | 37 (5.2%)  12 (1.7%) | 33 (18%)  8 (4.4%) | 17 (2.3%)  5 (0.7%) | 24 (11%)  7 (3.2%) | 32 (35%)  8 (8.8%) |
| *NA* | 0 (0%) | 9 (1.3%) | 2 (1.1%) | 2 (0.9%) | 2 (0.9%) | 1 (1.1%) |

*Notes.* BMI Body-Mass-Index; PBF Percent Body Fat; sd standard deviation; SMM Skeletal Muscle Mass; VFA Visceral Fat Area, NA no answer (information not provided by study participant)
