# Supplementary figures and images for: Evaluation of a validated questionnaire to assess the need for prevention or rehabilitation by preventive health examinations: a cross-sectional study of German employees aged 45 to 59 years (Ü45-check)
Source: Front Public Health. 2025 Jul 16;13:1480312. doi: 10.3389/fpubh.2025.1480312 (PMC12307417; doi:10.3389/fpubh.2025.1480312)

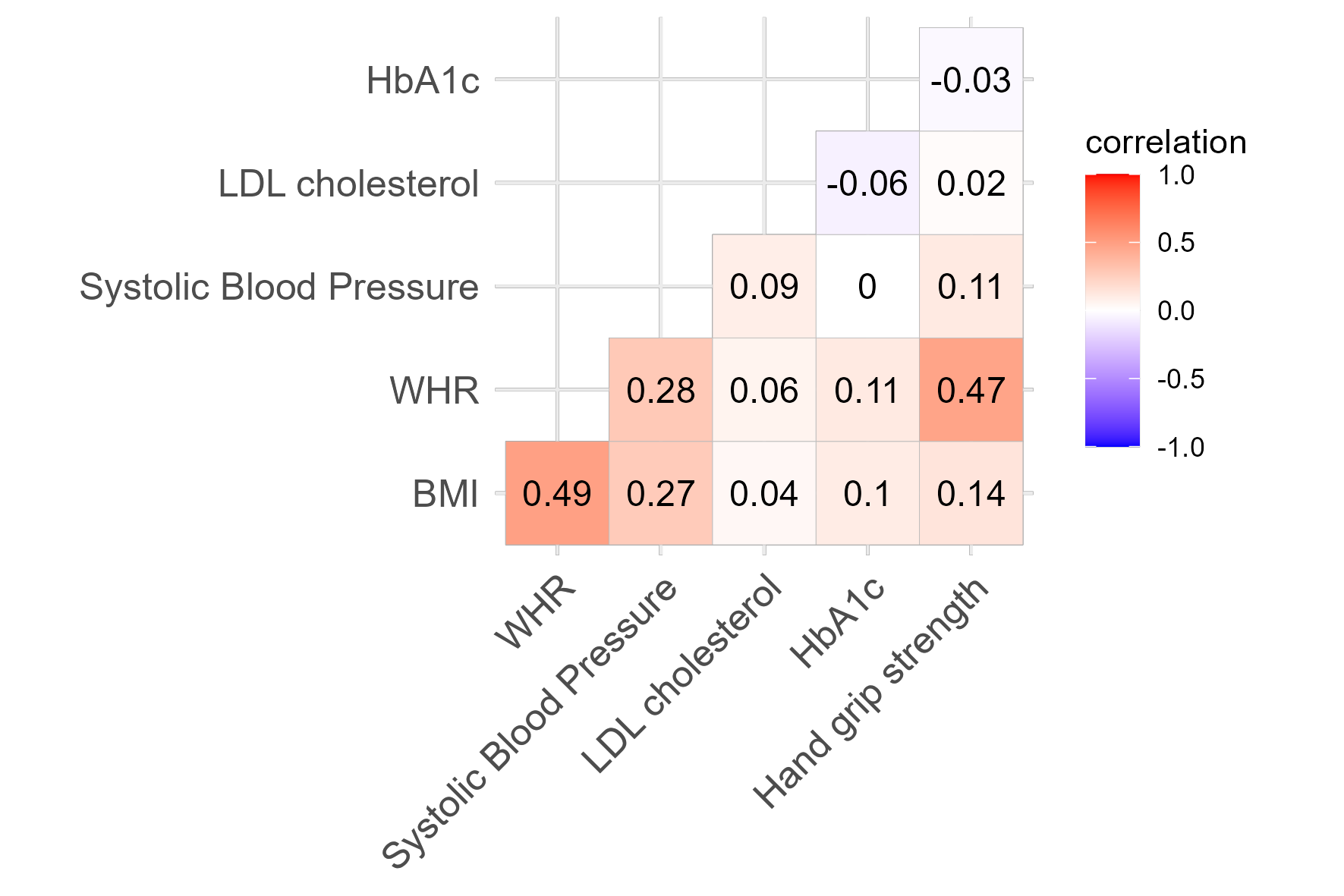

Supplement: Supplementary file 3 [file Image_1.tiff]
